# High-Quality Draft Genome Sequence of *Aneurinibacillus migulanus* ATCC 9999^T^ (DSM 2895), a Gramicidin S-Producing Bacterium Isolated from Garden Soil

**DOI:** 10.1128/genomeA.01227-15

**Published:** 2015-10-22

**Authors:** Jie-ping Wang, Bo Liu, Guo-hong Liu, Ci-bin Ge, Rong-feng Xiao, Xue-fang Zheng, Huai Shi

**Affiliations:** Agricultural Bio-Resources Research Institute, Fujian Academy of Agricultural Sciences, Fuzhou, Fujian, China

## Abstract

*Aneurinibacillus migulanus* ATCC 9999^T^ (DSM 2895) is a Gram-positive, round-spore-forming, and gramicidin S-producing bacterium. Here, we report the 6.35-Mb high-quality draft genome sequence of *A. migulanus* ATCC 9999^T^, which will provide useful information for the genomic taxonomy and phylogenomics of *Bacillus*-like bacteria.

## GENOME ANNOUNCEMENT

The *Bacillus*-like strain ATCC 9999^T^ (DSM 2895) was isolated by R. Synge from Moscow, Russia, and initially identified as a member of *Bacillus brevis* (now *Brevibacillus brevi*) (1). In 1993, the strain ATCC 9999^T^ was identified as a unique species and named as *Bacillus migulanus* sp. nov. (1). In 1996, however, *Bacillus migulanus* was reclassified as *Aneurinibacillus migulanus* comb. nov. belonging to the family *Paenibacillaceae* but not the family *Bacillaceae* (2). It is of great applicable significance that *A. migulanus* is a promising biocontrol agent against a range of plant-pathogenic bacteria and fungi, due to its ability to produce the antimicrobial peptide gramicidin S (3–5). Given the physiological properties and application prospects of *A. migulanus*, its type strain ATCC 9999^T^ was selected as one of the research objects in our “genome sequencing project for genomic taxonomy and phylogenomics of *Bacillus*-like bacteria.” Although two genome sequences of *A. migulanus* were reported (JYBN01000000 with 82 scaffolds and JYBO01000000 with 89 scaffolds) (4, 5), here, we presented the third draft genome sequence with higher quality (just 28 scaffolds).

The genome sequencing of *A. migulanus* ATCC 9999^T^ (DSM 2895) was performed via the Illumina Hieseq 2500 system. Two DNA libraries with insert sizes of 500 and 5,000 bp were constructed and sequenced. After filtering of the 1.14-Gb raw data, the 1.10-Gb clean data was obtained, providing approximately 200-fold coverage. The reads were assembled via the SOAPdenovo software version 1.05 (6), using a key parameter K setting at 71. Through the data assembly, 28 scaffolds with total length 6,348,994 bp were obtained, and the scaffold N_50_ was 4,944,581 bp. The average length of the scaffolds was 226,749 bp, and the longest and shortest scaffolds were 4,944,581 bp and 506 bp, respectively. A total of 91.49% clean reads could be aligned back to the genome, which covered 99.75% of the sequence.

The annotation of the genome was performed using the NCBI Prokaryotic Genomes Automatic Annotation Pipeline (PGAAP) (http://www.ncbi.nlm.nih.gov/genome/annotation_prok/) utilizing GeneMark, Glimmer, and tRNAscan-SE tools (7). A total of 6,273 genes were predicted, including 5,673 coding sequences (CDS), 504 pseudo genes, 85 tRNAs, and 12 rRNA genes. There were 3,938 and 2,984 genes assigned to COG and KEGG databases, respectively. The average DNA G+C content was 43.06%, agreeing with the values 43.04% of JYBN01000000 and 43.16% of JYBO01000000 (4, 5) but disagreeing slightly with the value 42.5 mol% acquired by HPLC determination (1).

### Nucleotide sequence accession numbers.

This whole-genome shotgun project has been deposited at DDBJ/EMBL/GenBank under the accession no. LGUG00000000. The version described in this paper is version LGUG01000000.
